# Longitudinal Associations Between Health-related Quality of Life and Female Service Member Readiness: Findings from the U.S. Millennium Cohort Study

**Published:** 2025-07-20

**Authors:** Isabel G. Jacobson, Sheila F. Castañeda, Yunnuo Zhu, Crystal L. Lewis, Felicia R. Carey

**Affiliations:** Deployment Health Research Department, Naval Health Research Center, San Diego, CA: Ms. Jacobson, Dr. Castañeda, Ms. Zhu, Dr. Lewis, Dr. Carey; Leidos, Inc., San Diego: Ms. Jacobson, Ms. Zhu, Dr. Lewis

The expansion of service women's occupational roles in the U.S. military has heightened focus on women's health, with the Department of Defense recently committing to “spending half a billion dollars each year on women's health research.” ^1^ These efforts could benefit from a comprehensive understanding of readiness among service women. Medical readiness may be broadly considered as the capability to achieve military mission and job success. ^2^ Given that readiness is multi-factorial and requires physical and mental fitness, measurable markers such as body mass index (BMI) and lost work days, while not exhaustive measures, are important to consider.

Body composition standards have been in place in the military for many decades ^3^ to ensure personnel readiness. ^4^ BMI is indirectly associated with retention in the military, as those who fail to meet weight standards are often separated from service. Although BMI cannot distinguish between fat and fat-free mass, a meta-analysis showed that BMI had a sensitivity of 51% and a specificity of 95% in women, suggesting that BMI performs well in correctly identifying those without obesity. ^5^ While those data also suggest that BMI is less accurate at identifying those with obesity, it may still offer utility as an initial screening for readiness, given its fast and non-invasive characteristics. Lost work days can also serve as an indicator of readiness, due to the rigors of military service, including deployments, posing frequent risks for injury, illness, or hospitalization, ^6^ leaving service members unable to perform their duties. Excessive lost work days may challenge mission completion and readiness.

Behavioral factors that may impede readiness include unhealthy sleep ^7^ and substance use; but screening for these factors may be cumbersome in fast-paced military environments. Measuring health-related quality of life (HRQOL)—or how mental, emotional, and physical capabilities affect daily functioning ^8^ —could provide a brief, non-intrusive screening tool for health-related factors associated with readiness. While HRQOL has been evaluated as a pre-dictor of health outcomes in service member and military spouse populations, ^9 , 10^ to our knowledge no studies have focused on U.S. service women. The aim of this analysis was to understand if HRQOL is significantly associated with subsequent readiness outcomes among active duty service women.

## Methods

Data were from active duty service women enrolled in the U.S. Millennium Cohort Study, the largest and longest-running study of military personnel and veterans. ^11^ Participants from all branches of service and components were enrolled in 5 panels: in 2001, 2004, 2007, 2011, and 2020. Among 260,228 enrolled participants, 79,872 were service women. ^11^

For this evaluation of baseline HRQOL and subsequent readiness outcomes, eligibility criteria included: enrollment in the first 4 panels (n=18,078 excluded from panel 5, as no follow-up survey was available for those participants at the time of this study); completion of the first follow-up survey (n=24,569 excluded who did not complete a follow-up survey); and serving on active duty at baseline and follow-up (n=16,426 excluded who were not on active duty at baseline; n=7,014 excluded who were not on active duty at follow-up). After application of all eligibility criteria, a total of 13,785 active duty service women were included in the study. HRQOL and covariates were reported at baseline, 2001-2011; readiness outcomes were assessed at first follow-up, 2004-2014.

Baseline HRQOL was coded from Veterans RAND 12 Item Short Form Survey (VR-12) summary scores using a validated scoring algorithm ^8^ to capture effects of somatic (PCS, or physical component summary) and emotional (MCS, or mental component summary) health problems on basic daily functioning, with higher scores indicating better HRQOL, and lower scores indicating worse HRQOL. The Veterans RAND 12 Item Health Survey was developed from the Veterans RAND 36 Item Health Survey, which was developed and modified from the original RAND version of the 36-item Health Survey version 1.0 (also known as MOS SF-36). ^12^

MCS and PCS scores have normative values, with a mean of 50 and standard deviation of 10, to compare with other U.S. populations. ^13^ Scores were categorized into 3 groups, using the 15th and 85th centiles to demarcate the low and high scoring groups, which roughly approximated 1 standard deviation from the mean. ^10^ Readiness-related outcomes included self-reported BMI and lost work days due to illness or injury (excluding time for pregnancy and childbirth).

The BMI readiness-related outcome was calculated from self-reported height and weight, dichotomized as women with a BMI under 30 kg/m ^2^ (i.e., more likely to be ready), versus those with a BMI of 30 kg/m ^2^ or greater (i.e., having obesity, less likely to be ready). The missed work days readiness-related outcome was calculated using the self-reported number of days that women were unable to work or perform usual activities within the past 3 years due to illness or injury; they were asked to exclude work days lost for pregnancy and childbirth. This outcome was dichotomized as those who missed 5 or fewer work days during the follow-up period (i.e., more likely to be ready) versus those who missed 6 or more days (i.e., less likely to be ready).

Socio-demographic and military covariates included age, marital status, race and ethnicity, pay grade, service branch, and enrollment panel. No collinearity was found when assessed among MCS and PCS scores and covariates, based on a variance inflation factor threshold of 4 or greater. Poisson regression models with robust error variance estimated prevalence ratios to assess the association between HRQOL and readiness outcomes, with adjustment for baseline covariates. All statistical analyses were conducted using SAS software version 9.4 (SAS Institute, Inc., Cary, NC). The study was approved by the Naval Health Research Center Institutional Review Board (NHRC.2000.0007).

## Results

Most service women in this sample were younger than age 35 years at baseline, non-Hispanic White race or ethnicity, and enlisted, while a plurality were married, in the Air Force, and enrolled in panel 1 (in 2001). Women with MCS scores less than 40.2 and PCS scores less than 47.3 were in the lowest 15th centile; those scoring greater than 57.9 and 58.0, respectively, were in the top 15th centile. Most women reported not having obesity (86.9%) and missing 5 or fewer workdays (66.2%) due to illness or injury (**Table** ).

**TABLE.** Active Duty Service Women Baseline Characteristics, Health-related Quality of Life Factors, and Follow-up Readiness Outcomes

Population Total	13,785	
Demographic and Military Characteristics at Baseline	No.	%
Age group, y		
17–24	4,912	35.6
25–34	6,526	47.3
35+	2,347	17.0
Race and ethnicity		
White, non-Hispanic	8,672	62.9
Black, non-Hispanic	2,747	19.9
Hispanic	1,069	7.8
Asian or Pacific Islander	837	6.1
Multi-racial	249	1.8
American Indian or Alaska Native	210	1.5
Missing	1	0
Marital status		
Single	5,269	38.2
Married	6,357	46.1
Other	2,158	15.7
Missing	1	0
Service branch		
Army	4,713	34.2
Navy	2,675	19.4
Marine Corps	406	2.9
Air Force	5,604	40.7
Coast Guard	387	2.8
Rank		
Enlisted	10,225	74.2
Officer	3,560	25.8
Enrollment panel		
Panel 1	5,079	36.8
Panel 2	2,751	20.0
Panel 3	3,235	23.5
Panel 4	2,720	19.7
HRQOL at baseline		
Mental HRQOL		
Lowest 15% (<40.2)	2,046	14.8
Middle 70% (40.2–57.9)	9,553	69.3
Highest 15% (<57.9)	2,047	14.8
Missing	139	1.0
Physical HRQOL		
Lowest 15% (<47.3)	2,056	14.9
Middle 70% (47.3–58.0)	9,592	69.6
Highest 15% (>58.0)	2,052	14.9
Missing	85	0.6
Readiness outcomes at follow-up		
Annual lost work days		
<=5 days	9,119	66.2
>6 days	4,181	30.3
Missing	485	3.5
Obesity status		
Non-obese	11,982	86.9
Obese	1,164	8.4
Missing	639	4.6

Abbreviations: No., number; y, years; HRQOL, health-related quality of life.

Adjusted multivariable models suggest that higher MCS and PCS scores were significantly associated with a higher likelihood for readiness, as defined by lack of obesity and fewer missed workdays ( **Figure** ). Women scoring in the top 15th centile for PCS demonstrated higher adjusted prevalences of non-obese BMI (APR 1.8, 95% CI 1.5, 2.2) and 5 or fewer lost workdays (APR 1.4, 95% CI 1.2, 1.5) compared to women scoring in the middle 70th centile. Results were similar for MCS scores, but measures of association were slightly lower.

**FIGURE.**
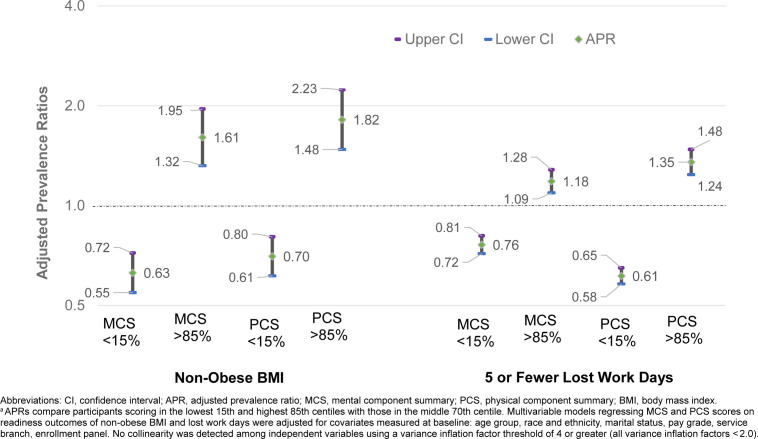
Adjusted Prevalence Ratios ^a^ for Readiness Outcomes by Mental and Physical Health-related Quality of Life Scores Among Active Duty U.S. Service Women

Results show a strong and consistent relationship between MCS and PCS scores in the lowest 15th centile and readiness outcomes. Women scoring in the lowest 15th centile for MCS demonstrated a lower adjusted prevalence of non-obese BMI (APR 0.6, 95% CI 0.6, 0.7) and 5 or fewer lost workdays (APR 0.8, 95% CI 0.7, 0.8). compared to women scoring in the middle 70th centile. These relationships were similar in magnitude and significance for women scoring in the lowest 15th centile for PCS.

## Discussion

Our findings suggest the VR-12 HRQOL instrument may be an efficient screening tool for health factors associated with readiness among service women. Low MCS and PCS scores were consistently associated with decreased likelihood of readiness (i.e., obese BMI and more lost work days). These relationships between HRQOL and readiness persisted after covariate adjustment, suggesting that HRQOL could stand alone as a brief screener for health-related readiness factors. Although these readiness-related outcomes could also be associated with subsequent HRQOL, this study's longitudinal design allowed a temporal assessment of HRQOL with each outcome, supporting a consistent relationship between these more global measures of health (MCS and PCS) and future readiness-related metrics.

The greatest magnitude of association for MCS and PCS was with BMI readiness outcome. As meeting weight standards in the military is tied to retention, this finding is critical to understanding service women's readiness. Research indicates that military service women who become pregnant may need additional support to sustain fitness during and after pregnancy. Recent research found that nearly 40% of active duty service women with a normal pregnancy (i.e., non-eclamptic) did not return to their baseline BMI after pregnancy. ^14^ The Marine Corps Artemis program, launched in 2021 at Camp Pendleton, ^14 , 15^ is designed to support women during and after pregnancy, but the program is limited to 1 service branch.

Additionally, the finding that both MCS and PCS scores are associated with BMI corroborates the proposition that there are mental and emotional components to weight control beyond simple caloric intake versus output. ^16^ Recent research utilizing Millennium Cohort Study data found that service members who screened positive for mental disorders such as post-traumatic stress disorder (PTSD) or depression were at higher risk for subsequent binge eating disorder. ^16^ Another study demonstrated that participants who screened positive for PTSD were more likely to experience subsequent weight gain. ^17^ A study of female veterans reported that military experiences including challenging food environments, sexual trauma, and pregnancy during service negatively affected eating behaviors. ^18^ Programs designed specifically with a holistic approach to women's weight management could be beneficial in helping them cope with military life stress. ^19^

Limitations of this study include the narrow definition of readiness, which may not fully capture all elements of readiness; however, BMI and missed work days are reasonable and objective proxies of duty fitness. MCS and PCS scores may have changed during the follow-up period due to unmeasured factors such as severe illness or injury. Although severe event prevalence is expected to be small, such factors may have biased results towards the null. Nonetheless, MCS and PCS provide global measures of physical and mental health. In fact, recent research on injury status and HRQOL observed MCS and PCS as stable over time, with baseline scores the strongest and most significant predictors of follow-up scores. ^20^

This report highlights the need for additional research to better understand female service member readiness, especially with renewed service focus on force lethality and deployability, and potential reviews of fitness and body composition standards. ^21^ Women-focused research on the unique needs of service women would fulfill a commitment to military women's health and, ultimately, result in a more ready female force.
